# Supplementary material for: Slow Evolution toward “Super-Aggregation” of the Oligomers Formed through the Swapping of RNase A N-Termini: A Wish for Amyloidosis?
Source: Int J Mol Sci. 2022 Sep 23;23(19):11192. doi: 10.3390/ijms231911192 (PMC9569824; doi:10.3390/ijms231911192)
Supplement: Supplementary file 1 [file ijms-23-11192-s001.zip › ijms-1889203-supplementary.pdf]

Supplementary data for

Slow evolution toward "super-aggregation" of the oligomers formed through the swapping of RNase A N-termini: a wish for amyloidosis?

by

G. Gotte[1*#], E. Butturini[1*], I. Bettin[1], I. Noro[1], A. Mahmoud-Helmy[1], A. Fagagnini[1], B. Cisterna[2] and M. Malatesta[2]

[1] *Department of Neuroscience, Biomedicine & Movement Sciences, Biological Chemistry Section, University of Verona, Strada Le Grazie 8, I-37134 Verona, Italy.*
[2] *Department of Neuroscience, Biomedicine & Movement Sciences Anatomy & Histology Section, University of Verona, Strada Le Grazie 8, I-37134 Verona, Italy.*

*These two authors contributed equally to the work

[#] Corresponding author

The data included are the Supplementary Figures S1-S3

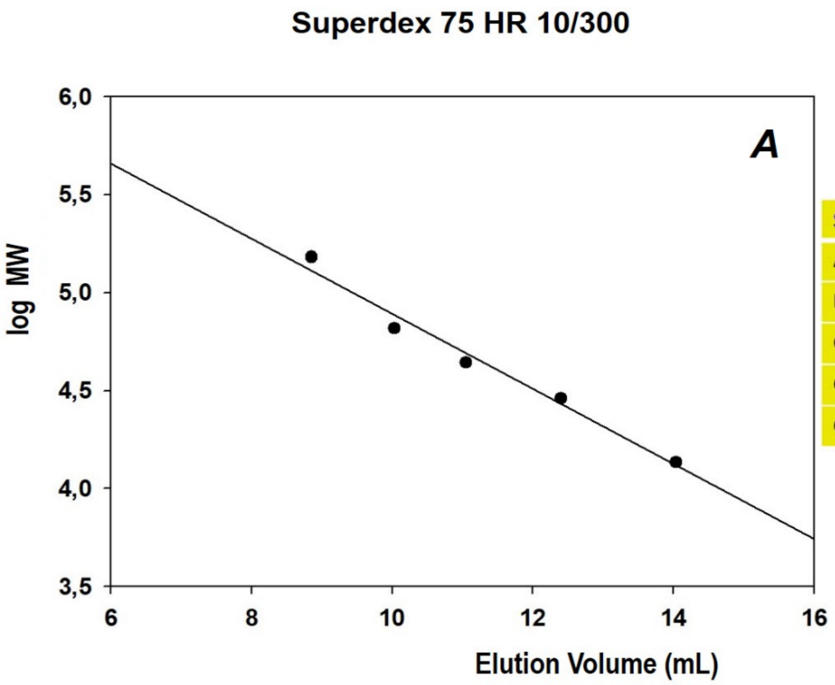

STD proteins	MW (kDa)	Log MW	Elut. Vol. (mL)
Alcohol Dehydrogen.	150	5.176	8.54
Bovine Seralbumin	66	4.820	9.72
Ovalbumin	45	4.653	10.94
Carbonic Anhydrase	29	4.462	12.21
Cytochrome C	12.4	4.094	14.08

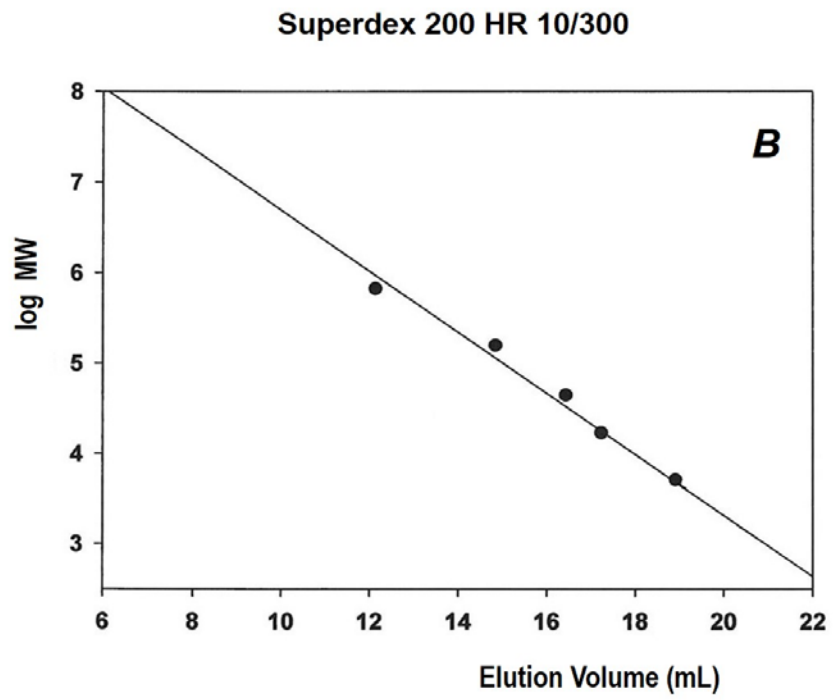

STD proteins	MW (kDa)	Log MW	Elut. Vol. (mL)
Thyroglobulin	670	5.826	11.87
Alcohol Dehydrogen.	150	5.176	14.60
Ovalbumin	45	4.653	16.04
Cytochrome C	12.4	4.094	17.22
Aprotinin	6.5	3.813	18.92

Figure S1. Calibration curves of the two Superdex 75 and 200 H10/300 SEC columns (**A** & **B**, respectively) used to purify and analyse the SAs produced by RNase A. The relative standard (STD) proteins data are reported in each table flanking the calibration curves.

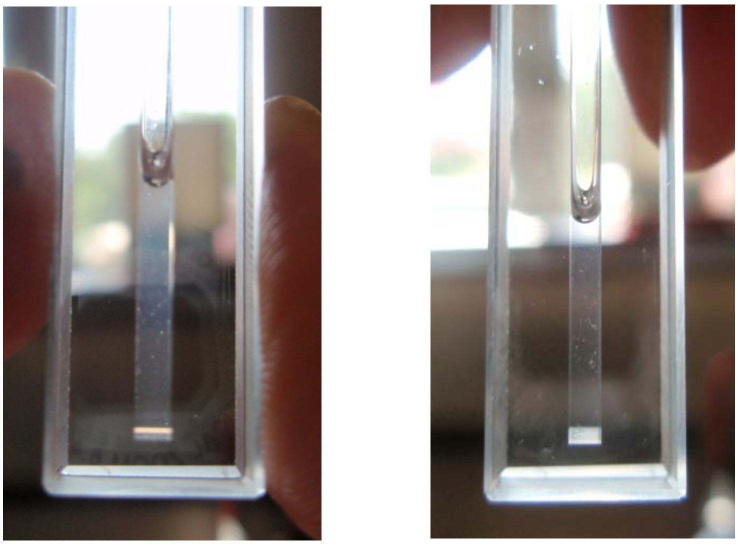

Figure S2. Turbidity of RNase A SAs: **Left**: quartz cuvette containing the SA-2 sample recovered from the Superdex 75 HR 10/300 SEC column and concentrated with Amicon-3 ultrafilters. **Right**: the same sample after being centrifuged at 14’500 rpm for 8 min in a Mini-Eppendorf centrifuge to partly eliminate turbidity.

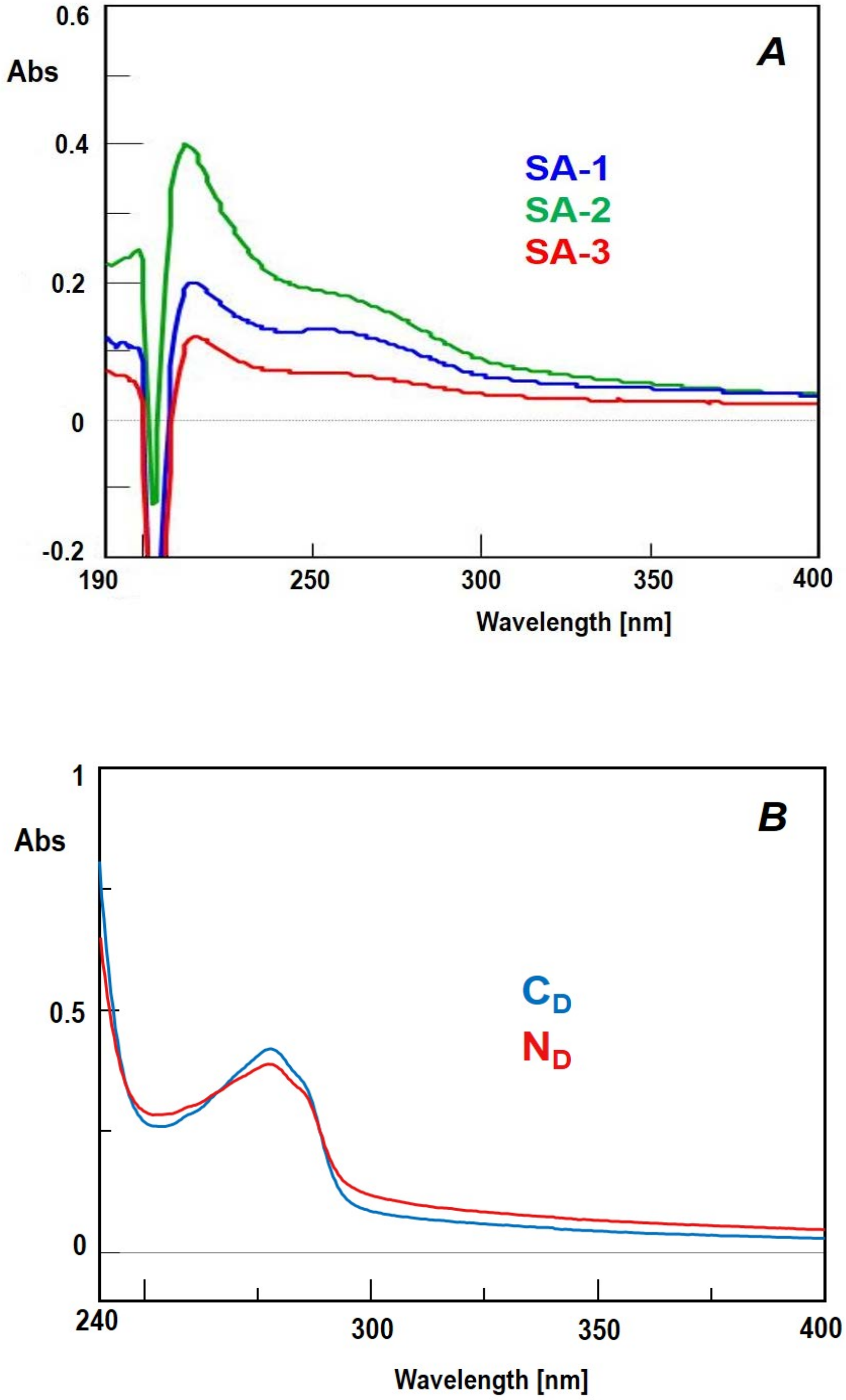

Figure S3. UV-vis spectra of RNase A SAs (**A**) and of N_Ds and C_Ds (**B**) produced upon 40 % HAc lyophilization and purified through SEC (see Figure 1 & 2). Each color-code is reported inside.
